# Relationship between mediation analysis and the structured life course approach

**DOI:** 10.1093/ije/dyw254

**Published:** 2016-10-06

**Authors:** Laura D Howe, Andrew D Smith, Corrie Macdonald-Wallis, Emma L Anderson, Bruna Galobardes, Debbie A Lawlor, Yoav Ben-Shlomo, Rebecca Hardy, Rachel Cooper, Kate Tilling, Abigail Fraser

**Affiliations:** 1 MRC Integrative Epidemiology Unit; 2 School of Social and Community Medicine, University of Bristol, Bristol, UK and; 3 MRC Unit for Lifelong Health and Ageing at UCL, University College London, London, UK

## Abstract

Many questions in life course epidemiology involve mediation and/or interaction because of the long latency period between exposures and outcomes. In this paper, we explore how mediation analysis (based on counterfactual theory and implemented using conventional regression approaches) links with a structured approach to selecting life course hypotheses. Using theory and simulated data, we show how the alternative life course hypotheses assessed in the structured life course approach correspond to different combinations of mediation and interaction parameters. For example, an early life critical period model corresponds to a direct effect of the early life exposure, but no indirect effect via the mediator and no interaction between the early life exposure and the mediator. We also compare these methods using an illustrative real-data example using data on parental occupational social class (early life exposure), own adult occupational social class (mediator) and physical capability (outcome).

**Key Messages**

- We have explored with theory and simulation studies which mediation and interaction parameters are implied by each of a set of commonly used life course hypotheses, thus showing the links between these methods.
- As demonstrated in previous studies, mediation methods based on counterfactual theory have advantages over conventional mediation analysis, including the ability to incorporate exposure-mediator interactions, deal with measured intermediate confounding (confounders of the mediator-exposure relationship that are also descendents of the exposure) and non-linear relationships.
- Mediation analysis and the structured life course approach are linked in that alternative life course hypotheses suggest the presence of differing sets of mediation and interaction parameters, but the approaches define parameters with different interpretations, and using both mediation analysis (choosing between conventional mediation analysis and the counterfactual-based approaches based on the importance of the factors in the previous point) and the structured life course approach in parallel may therefore be informative.
- Conventional mediation analysis, mediation analysis based on counterfactual theory, and the structured life course approach all share a common set of assumptions, including no measurement error/misclassification bias and no unmeasured confounding of the effects of exposure on outcome or mediator on outcome.

## Introduction

Life course epidemiology seeks to understand how factors across the life course influence health.^1^ Many life course analyses can be framed as questions about mediation – the study of pathways linking an exposure to an outcome. In this Education Corner, we evaluate the relationship between mediation analyses and a method designed to compare alternative hypotheses for how life course exposures combine to affect an outcome (‘structured life course approach’).

### Mediation analysis

Conventionally, mediation analyses are performed by regressing the outcome on the exposure with and without the mediator(s).^2^ The unadjusted (or confounder-adjusted) estimate is referred to as the ‘total effect’ of the exposure on the outcome, the mediator-adjusted estimate is referred to as the ‘direct effect’ [the effect of the exposure on the outcome that is not mediated by the mediator(s) included in the model] and the difference between them is the ‘indirect effect’ [the effect of the exposure on the outcome that acts through the mediator(s) included in the model]. This approach, although widely used, has limitations including: (i) susceptibility to bias when the mediator is measured with error;^3–6^ (ii) inability to derive estimates of interactions between the exposure and mediator; (iii) collider bias^7^^,^^8^ may be induced by conditioning on the mediator in the presence of unmeasured mediator-outcome confounders; (iv) a direct effect cannot be estimated if a descendent (consequence) of the exposure confounds the mediator-outcome effect (‘intermediate confounding’); and (v) the method is only approximate for binary outcomes or mediators.^9^ Recent work has developed alternative approaches to the study of mediation based on counterfactual theory, which address some of these limitations. A full review of these methods is beyond the scope of this Education Corner, and such reviews are available elsewhere,^6^^,^^10^ but a few key points are worth noting. The counterfactual approach to mediation defines effects in terms of counterfactuals, i.e. it is a non-parametric approach, in contrast to the conventional approach which is based on linear regression parameters. That said, under certain settings and assumptions, the mediation effects defined by counterfactuals can be estimated using regression methods.^11^ Key advantages of these novel approaches over the conventional approach are that they lead to counterfactual definitions of mediation parameters that can be estimated, they can estimate mediation parameters in the presence of exposure-mediator interactions^12^ and they adjust for measured intermediate confounders.^13^

The ability to incorporate measured intermediate confounders within mediation analyses using the counterfactual-based approaches is likely to be important in many life course applications. With an exposure measured during early life and a mediator measured during adulthood, it is quite likely that there may be factors caused by the early life exposure that confound the effect of the mediator on the outcome.

Consideration of exposure-mediator interactions is also important within life course epidemiology; for example, there is a large evidence base demonstrating interactions between low birthweight and later adiposity with respect to later cardiometabolic health.^14^ Within the conventional mediation analysis framework, it is simply not possible to estimate mediation parameters (direct and indirect effects) in the presence of an exposure-mediator interaction. The parameters that can be estimated are the effect of the exposure on the outcome across strata of the mediator and/or the effect of the mediator on the outcome across strata of the exposure. Thus the ability to fully combine mediation and interaction analyses and to define direct and indirect effects within the counterfactual framework is a key strength of these methods for life course analyses. The term ‘interaction’ implies two interventions, whereas ‘effect modification’ refers to the effect of one intervention varying across strata of a second, not necessarily causal, variable.^15^ For the purposes of this paper, since we are assuming causal effects of both the exposure and mediator on the outcome, we will use the term interaction.

Here we focus on two types of mediation parameters that can be defined and estimated using counterfactual-based mediation approaches: controlled or natural/pure. A controlled direct effect is defined as the effect of an exposure on an outcome while holding the mediator constant at a given value. In contrast, a natural direct effect fixes the mediator to the level that would have occurred ‘naturally’ for a given individual, i.e. for a binary exposure, the level of mediator is fixed to the value each individual would have experienced in the absence of exposure. Controlled and natural direct effects are equal in the absence of exposure-mediator interaction. Further discussion of the differences between these mediation parameters can be found elsewhere.^16^

### Structured life course approach

Within life course epidemiology, we are often interested in comparing how an exposure measured at multiple time points across the life course combine to influence an outcome. For example, one might hypothesize a ‘critical period’, i.e. a specific window of time during which an exposure has a lasting and irreversible impact on an outcome. Alternatively, it may be hypothesized that the effect of an exposure across the life course is cumulative, i.e. the risk of the outcome rises in proportion to to the duration of exposure. The structured life course approach is typically used when the interest lies in assessing the role of a single characteristic measured at multiple points in the life course; within the mediation framework, the earlier measure can be considered the exposure and the later measure can be considered the mediator. It is also worth noting that the life course models refer only to the variables included in the analysis, such that for example a critical period model does not preclude the existence of other mediators on the causal pathway from exposure to outcome. A full description of potential life course models has been published elsewhere.^1^^,^^17^

Recently, a structured modelling approach for defining and comparing alternative life course hypotheses has been developed.^18^^,^^19^ This approach estimates statistical models that correspond to each alternative hypothesis; for example in a critical period model, the outcome would be regressed only on an indicator of exposure in that critical period since exposure at all other time points is assumed to have no effect on the outcome. In contrast, an accumulation model would regress the outcome on the number of occasions at which an individual experiences the exposure of interest. The set of models corresponding to life course hypotheses of interest are then compared. The paper initially proposing this method used an F test to compare each of the models with a saturated model and hence to select the hypothesis that best matched the observed data.^19^ Recent work has extended the approach using the least absolute shrinkage and selection operator (lasso) to select the hypothesis or set of hypotheses that explain the most amount of variance in the outcome. In simulation studies, the lasso approach identified the most suitable hypothesized model with high probability in moderately sized samples, but with lower probability for mobility hypotheses (e.g. a hypothesis that states that those who moved from high social class in childhood to low social class in adulthood would have the worst outcome; in other words, a hypothesis including an interaction between time-specific exposures) or highly correlated exposures.^18^ The authors also compared alternative approaches to lasso, i.e. F tests and the Akaike information criterion, and showed that these alternative methods did not identify the correct hypothesis as often and were more likely to favour compound hypotheses over simple ones (in contrast to lasso). The lasso method is extremely flexible, enabling consideration of a wide variety of potential life course hypotheses, including models specifying interactions or non-linear associations. The lasso approach can be thought of as one step towards exploration of causal effects, and further steps are necessary before reaching firm causal conclusions, for example: consideration of sources of bias and unmeasured confounding; replication in other datasets (including with different distributions of exposures and mediators); and use of other methods to interrogate further the selected hypotheses (e.g. marginal structural models).

### Rationale for comparing mediation analysis and the structured life course approach

Mediation analyses and the structured life course approach are two alternative ways of approaching life course research questions. The approaches ask different questions: the first attempts to quantify the degree to which an exposure-outcome effect is explained by a mediating variable, whereas the second seeks to identify which life course hypotheses explain the most variance in an outcome. However, the two approaches are mathematically linked. The alternative life course hypotheses have different implications in terms of the presence of mediation and exposure-mediator interactions. Examining these methods side by side will help to improve understanding of the links between mediation and interaction parameters and life course hypotheses, enabling clearer insight into the use of both methods within life course epidemiology and facilitating comparisons of multiple studies addressing the same life course question but using different analytical approaches.

In this Education Corner, we illustrate the links between mediation analysis and the structured life course approach using five simulated scenarios, with supportive analysis of data from a prospective cohort to show effects of occupational social class across the life course on physical capability (the ability to perform the physical tasks of daily living; a key marker of healthy ageing).^20–22^ Previous studies have shown that low socioeconomic position (SEP) is associated with poorer physical capability,^23–27^ with evidence that associations between childhood SEP and physical capability in adulthood are partly, but not fully, mediated by adult SEP.^23^

## Methods

### Simulated data scenarios

**Figure 1.**
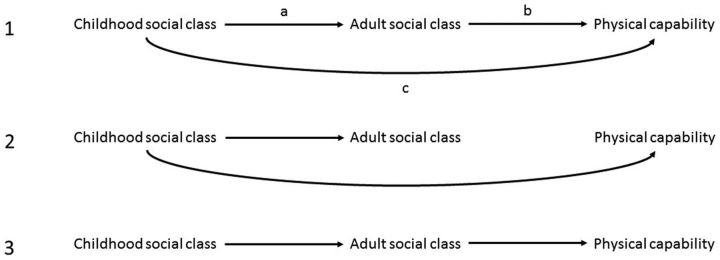
Schematic diagram of hypothesized relationships between social class and physical capabilityScenario 1: both childhood and adult social class influence physical capability, with partial but not full mediation of the childhood social class-physical capability effect by adult social class. The direct effect of childhood social class on physical capability is represented by arrow c, and the indirect effect via adult social class (childhood social class-adult social class-physical capability; paths a-b) is estimated in our analyses as the difference between the total and direct effects. Scenario 2: childhood social class influences physical capability, with no mediation through adult social class (i.e. adult social class does not affect physical capability). Scenario 3: adult social class influences physical capability with no direct effect of childhood social class (i.e. childhood social class influences physical capability only through its effect on adult social class). Scenarios 4 and 5 include an interaction term within our additive model for the outcome, which cannot easily be shown on a DAG since causal diagrams are non-parametric. These scenarios reflect the first scenario, with the addition of an interaction term that is in the opposite direction to the main effects of childhood and adult social class in relation to the outcome (scenario 4), or in the same direction (scenario 5).

We simulated five datasets, each with a binary exposure, binary mediator and continuous outcome, with a sample size of 20 000 in each case. For consistency with our real-data example, we refer to these as childhood social class (exposure), adult social class (mediator) and physical capability (outcome). For both childhood and adult social class, low social class (exposed) is coded as 1 and high social class (unexposed) is coded as 0. The five scenarios are: 1: both childhood and adult social class affect physical capability, with partial mediation of the childhood social class-physical capability effect by adult social class 2: childhood social class affects physical capability, with no mediation through adult social class (i.e. childhood social class influences physical capability only through its effect on adult social class); 3: adult social class affects physical capability with no direct effect of childhood social class (i.e. childhood social class influences physical capability only through its effect on adult social class); 4: both childhood and adult social class affect physical capability, with an interaction such that people of low social class in both childhood and adulthood have better physical capability than would be predicated based on the inverse independent effects of each in this additive model (i.e. the interaction term is in the opposite direction to the main effects of childhood and adult social class); and 5: both childhood and adult social class affect physical capability, with an interaction term in the same direction as the main effects of childhood and adult social class. The statistical code for the simulations is shown in the Appendix (available as Supplementary Data at *IJE* online), and directed acyclic graphs illustrating the scenarios are shown in Figure 1.

### Real-data example

The Avon Longitudinal Study of Parents and Children (ALSPAC) is a prospective cohort study that recruited 14 541 pregnant women in 1991–92.^28^ The study website contains details of all the data and a fully searchable data dictionary: [http://www.bris.ac.uk/alspac/researchers/data-access/data-dictionary/]. Ethical approval was obtained from the ALSPAC Law and Ethics Committee and the local research ethics committees. The mothers from this cohort are the participants in our analysis. Women's childhood and adult social class (both dichotomized to high, coded as 0, and low, coded as 1) were self-reported; details are shown in the Appendix 9 (available as Supplementary Data at *IJE* online). Approximately 20 years after recruitment, 2893 women attended a follow-up clinic [mean age 50.8 years, standard deviation (SD) 4.4 years] where they completed a grip strength test, a timed standing balance test, a timed chair rise test and a timed 3-m walk. These measures were each re-scaled to take values 0–1 (with people unable to perform the test due to health reasons assigned a value of 0), and subsequently summed into an overall index of physical capability that took values ranging from 0 (low capability) to 4 (high capability). The physical capability score was regressed on age at assessment; the residuals of this regression were used as an age-adjusted measure of physical capability as the outcome in these analyses; full details are shown in the Appendix (available as Supplementary Data at *IJE* online).

### Statistical approaches

The following methods were applied to each simulated dataset and the ALSPAC data.

#### Conventional mediation and interaction analysis

We performed linear regressions of the physical capability score on: (i) childhood social class (‘total effect’ of childhood social class); (ii) childhood social class adjusted for adult social class (‘direct effect’ of childhood social class); and (iii) childhood and adulthood social class and the interaction between them. The third approach is included as a demonstration of how the assumption of no exposure-mediator interaction could be tested, and as an illustration of the parameters that can be estimated in conventional analyses when such an interaction is present. Given that the mediator in our example is binary, we have used the difference method to estimate the indirect effect (total effect–direct effect effect). Confidence intervals for the indirect effects were obtained through bootstrapping with 100 replications. Further discussion of the difference method versus the product method for calculating indirect effects can be found elsewhere.^9^^,^^29^

#### Four-way decomposition of mediation and interaction

Four-way decomposition analysis,^12^ which enables decomposition of a total effect into a controlled direct effect [CDE(m), i.e. when the mediator M = m], a pure indirect effect (PIE), a reference interaction (INT_ref_) and a mediated interaction (INT_med_), was selected as an example of mediation analysis methods in the presence of exposure mediator interaction, based on counterfactual theory. The interpretation of the four parameters defined by this method is provided in Box 1, which is adapted from the paper that reported on the development of this approach.^12^ Further discussion of the interpretation of the ‘mediated interaction’ parameter has also recently been published.^30^ In Table 2 and the Appendix (available as Supplementary Data at *IJE* online) we outline the relationship between mediation analysis and the structured life course approach.

**Box 1.** Parameters and their interpretation defined by the four-way decomposition analysis

Parameter	Interpretation	Counterfactual definition*
Controlled direct effect [CDE(0)]	Due to neither mediation nor interaction	E(*Y*_10_ – *Y*_00_)
Reference interaction (INT_ref_)	Due to interaction only	E(*Y*_11_ – *Y*_10_ – *Y*_01_ + *Y*_00_)*M*_0_
Mediated interaction (INT_med_)	Due to mediation and interaction	E(*Y*_11_ – *Y*_10_ –*Y*_01_ + *Y*_00_)(*M*_1_ – *M*_0_)
Pure indirect effect (PIE)	Due to mediation only	E(*Y*_01_ – *Y*_00_)(*M*_1_ – *M*_0_)

*Where *Y_xm_* is the value of the outcome (Y) if the exposure (X) were set to *x* and mediator (M) were set to *m*, and *M_x_* is the value of the mediator if the exposure were set to *x.* The formulae given are only valid for binary exposure and binary mediator and are re-formulation of VanderWeele's ‘empirical analogs’.^12^

#### A structured approach to testing life course hypotheses

The structured life course approach uses a model selection procedure to identify, from a set of pre-defined hypotheses for the relationships between exposure variables and outcome, the hypothesis that explains the greatest amount of variation in the outcome.^19^ For completeness in our comparisons between mediation analysis and the structured life course approach, we considered a broad range of potential hypotheses: early life critical period (only childhood social class influences physical capability); adult critical period (only adult social class influences physical capability); accumulation (childhood and adult social class have equal magnitudes of effect on physical capability in a mutually adjusted model); increasing social class (the only difference in physical capability is for people who moved from low social class in childhood to high social class in adulthood, ‘upward mobility’); decreasing social class (the only difference in physical capability is for people who moved from high social class in childhood to low social class in adulthood, ‘downward mobility’); always exposed (people who have low social class in both childhood and adulthood have a difference in physical capability compared with all other groups); and ever exposed (there is a difference in physical capability between people who were high social class in both childhood and adulthood compared with those who had low social class in childhood, adulthood or both). The two critical period models assume that timing of exposure rather than duration is key. The ‘exposure scores’ implied by each of the life course hypotheses are outlined in Box 2. We used a procedure based on the least absolute shrinkage and selection operator (lasso) to identify which of the above hypotheses explained the greatest amount of variation in physical capability, allowing for the possibility that more than one hypothesis may be working in combination.^18^ The first hypothesis selected is the one that explains the highest proportion of variation in the outcome. A covariance test is used to assess whether adding a second hypothesis improves the model fit.^31^

**Box 2.** Levels of exposure under the alternative life course models tested by the structured approach

Life course social class* pattern**	Life course hypothesis***						
	Early life critical period	Adult critical period	Accumulation	Increasing social class	Decreasing social class	Always exposed	Ever exposed
High-high	0	0	0	0	0	0	0
High-low	0	1	1	0	1	0	1
Low-high	1	0	1	1	0	0	1
Low-low	1	1	2	0	0	1	1

*Social class is coded as 1 for low and 0 for high.

**Social class in childhood to social class in adulthood.

***Values imply the ‘exposure score’ that participants with each life course pattern would be assigned under each hypothesis; e.g. under early life critical period model, those who experience a low-high pattern are expected to have the same exposure level (and hence the same mean value for the outcome) as those who experienced a low-low pattern.

Conventional regression analyses and the four-way decomposition analysis were performed in Stata,^32^ and the lasso analysis for the structured life course approach was performed in R.^33^ Statistical code for the four-way decomposition analysis is provided in the Appendix (available as Supplementary Data at *IJE* online); statistical code suitable for the structured life course approach can be found elsewhere.^18^

## Results

The mediation and interaction parameters corresponding to each life course hypothesis assessed using the structured approach are shown in Table 1 for conventional mediation analysis and Table 2 for four-way decomposition analyses. For example, an early life (exposure) critical period model suggests a direct effect only, whereas an adult (mediator) critical period model suggests only an indirect effect of the early life exposure (no direct effect) provided there is an effect of early life on later life exposures. In contrast, the ‘always exposed’ hypothesis implies the presence of only the interaction terms of the four-way decomposition (reference and mediated interaction), whereas the ever exposed hypothesis suggests all four mediation and interaction terms of the four-way decomposition are non-zero. The derivation of these relationships is presented in the Appendix (available as Supplementary Data at *IJE* online). Note that we assume the presence of an effect of the exposure on the mediator, which is not explicitly tested in the structured life course approach.

**Table 1.** Relationship between mediation analysis and the structured life course approach

If the structured hypothesis contains a term for:	Provides evidence of:	
Early life critical period	Direct effect	
Adult critical period		Indirect effect*
Accumulation	Direct effect	and Indirect effect*
Increasing social class	Direct effect	and Indirect effect*
Decreasing social class	Direct effect	and Indirect effect*
Always exposed	Direct effect	and Indirect effect*
Ever exposed	Direct effect	and Indirect effect

If the structured hypothesis contains more than one term, then these could cancel each other out in certain circumstances.

In our example, the exposure is effect of childhood social class (X), the outcome is physical capability (Y) and the mediator is adult social class (M). See Figure 1 for a diagram of hypothesized relationships between social class and physical capability.

*Provided there is an effect of exposure on mediator

**Table 2.** Relationship between VanderWeele's four-way decomposition for mediation and interaction and the structured life course approach

If the structured hypothesis contains a term for:	Provides evidence of: 4-way decomposition			
Early life critical period	CDE(0)			
Adult critical period				PIE*
Accumulation	CDE(0)			PIE*
Increasing social class	CDE(0)	INT_ref_	INT_med_*	
Decreasing social class		INT_ref_	INT_med_*	PIE*
Always exposed		INT_ref_	INT_med_*	
Ever exposed	CDE(0)	INT_ref_	INT_med_*	PIE*

Other decompositions using recombinations of the 4-way decomposition are possible.^13^ If the structured hypothesis contains more than one term, then these could cancel each other out in certain circumstances. In our example, the exposure is effect of childhood social class (X), the outcome is physical capability (Y) and the mediator is adult social class (M). See Figure 1 for a diagram of hypothesized relationships between social class and physical capability.

*Provided there is an effect of exposure on mediator.

### Simulation study results

The expected mean level of the outcome according to each life course pattern of social class for each of the simulated datasets is shown in Box 3. In scenario 1 (both childhood and adult social class influence physical capability, with partial mediation), the conventional mediation analysis (Table 3) and four-way decomposition analysis (Table 4) confirmed the presence of both a direct effect of childhood social class and an indirect effect via adult social class, with no strong evidence of an interaction. The structured life course approach (Table 5) supported accumulation and adult critical period hypotheses, which Tables 1 and 2 suggest is compatible with the mediation analysis: a combination of an accumulation hypothesis and an adult critical period model suggests that both a CDE(0) and PIE are present in the four-way decomposition analysis.

**Box 3.** Expected mean outcome value in simulated datasets according to exposure pattern

	Scenario 1	Scenario 2	Scenario 3	Scenario 4	Scenario 5
Life course social class pattern*					
High-high	2.45	2.22	2.22	2.45	2.45
High-low	1.95	2.22	1.97	1.95	1.95
Low-high	2.20	1.97	2.22	2.20	2.20
Low-low	1.70	1.97	1.97	1.90	1.40

*Social class in childhood to social class in adulthood.

**Table 3.** Effects of social class across the life course and physical capability using conventional regression analyses within five simulated scenarios (*N* = 20 000) and the real-data example (*N* = 2122)

Effect of interest	Mean difference (95% CI) in physical capability score*					
	Scenario 1**	Scenario 2**	Scenario 3**	Scenario 4**	Scenario 5**	Real-data example
‘Total effect’ of low social class in childhood	−0.45	−0.25	−0.10	−0.36	−0.61	−0.09
	(−0.47 to −0.44)	(−0.27 to −0.24)	(−0.12 to −0.09)	(−0.38 to −0.34)	(−0.63 to −0.59)	(−0.13 to −0.06)
‘Direct effect’ of low social class in childhood	−0.25	−0.25	0.00	−0.22	−0.32	−0.07
	(−0.27 to −0.24)	(−0.27 to −0.24)	(−0.01 to 0.02)	(−0.24 to −0.20)	(−0.34 to −0.29)	(−0.11 to −0.04)
Indirect effect of low social class in childhood via adult social class	−0.20	0.00	−0.10	−0.14	−0.29	−0.02
	(−0.21 to −0.19)	(−0.01 to 0.01)	(−0.11 to −0.10)	(−0.15 to −0.13)	(−0.31 to −0.28)	
**Analysis including an interaction between childhood and adulthood social class** **						
Effect of low social class in childhood on physical capability						
a) in those who were low social class in adulthood	−0.23	−0.23	−0.01	−0.07	−0.53	−0.01
	(−0.26 to −0.19)	(−0.26 to −0.19)	(−0.05 to 0.02)	(−0.14 to 0.01)	(−0.61 to −0.45)	(−0.08 to 0.06)
b) in those who were high social class in adulthood	−0.26	−0.26	0.01	−0.25	−0.27	−0.10
	(−0.27 to −0.24)	(−0.28 to −0.24)	(−0.01 to 0.02)	(−0.27 to −0.24)	(−0.29 to −0.25)	(−0.14 to −0.05)
Effect of low social class in adulthood and physical capability						
a) in those who were low social class in childhood	−0.49	0.01	−0.26	−0.30	−0.78	−0.07
	(−0.51 to −0.47)	(−0.01 to 0.03)	(−0.28 to −0.24)	(−0.33 to −0.26)	(−0.81 to −0.75)	(−0.12 to −0.03)
b) in those who were high social class in childhood	−0.52	−0.02	−0.25	−0.48	−0.52	−0.16
	(−0.56 to −0.49)	(−0.06 to 0.01)	(−0.28 to −0.21)	(−0.52 to −0.45)	(−0.56 to −0.48)	(−0.23 to −0.09)
*P*-value for interaction between childhood and adult social class	0.13	0.10	0.38	<0.001	<0.001	0.03

*Physical capability score is an age-adjusted measure that can take values 0–4, with higher values indicating better physical capability.

**Scenario 1: effect of low social class in both childhood and adulthood on (lower) physical capability, with partial mediation. Scenario 2: effect of social class in childhood on physical capability, with no mediation by adult social class. Scenario 3: effect of social class in childhood on physical capability, with complete mediation by adult social class. Scenario 4: effects of low social class in both childhood and adulthood on (lower) physical capability, with an interaction term that is in the opposite direction to the main effects of childhood and adult social class. Scenario 5: effects of low social class in both childhood and adulthood on (lower) physical capability, with an interaction in the same direction as the main effects of childhood and adult social class.

**Table 4.** Four-way decomposition (estimate, 95%confidence interval) of the relationship between social class and physical capability into mediation and interaction parameters within five simulated scenarios (*N* = 20 000) and the real-data example (*N* = 2122)

	Scenario 1*	Scenario 2*	Scenario 3*	Scenario 4*	Scenario 5*	Real-data example
Total effect of low social class in childhood	−0.45	−0.25	−0.10	−0.36	−0.61	−0.09
	(−0.47 to −0.44)	(−0.27 to −0.24)	(−0.12 to −0.09)	(−0.38 to −0.34)	(−0.63 to −0.59)	(−0.13 to −0.06)
Controlled direct effect	−0.26	−0.26	0.01	−0.25	−0.27	−0.10
	(−0.27 to −0.24)	(−0.28 to −0.24)	(−0.01 to 0.02)	(−0.28 to −0.23)	(−0.29 to −0.25)	(−0.14 to −0.05)
Proportion attributable	0.57	1.03	−0.05	0.71	0.44	1.03
	(0.54 to 0.59)	(0.99 to 1.07)	(−0.22 to 0.12)	(0.67 to 0.75)	(0.41 to 0.47)	(0.78 to 1.28)
Reference interaction	0.003	0.003	−0.002	0.02	−0.02	0.02
	(−0.001 to 0.006)	(−0.001 to 0.01)	(−0.005 to 0.002)	(0.01 to 0.02)	(−0.03 to −0.02)	(0.002 to 0.03)
Proportion attributable	0.01	−0.01	0.01	−0.04	0.03	−0.19
	(−0.01 to 0.002)	(−0.02 to 0.002)	(−0.02 to 0.05)	(−0.06 to −0.03)	(0.03 to 0.04)	(−0.38 to −0.002)
Mediated interaction	0.01	0.01	0.01	0.08	−0.11	0.02
	(−0.004 to 0.03)	(−0.003 to 0.03)	(−0.02 to 0.01)	(0.05 to 0.10)	(−0.13 to −0.08)	(0.001 to 0.04)
Proportion attributable	−0.03	−0.06	0.07	−0.21	0.17	−0.20
	(−0.06 to 0.01)	(−0.12 to 0.01)	(−0.09 to 0.23)	(−0.28 to −0.14)	(0.14 to 0.21)	(−0.39 to −0.003)
Pure indirect effect	−0.21	−0.01	−0.10	−0.19	−0.21	−0.03
	(−0.23 to −0.20)	(−0.02 to 0.01)	(−0.11 to −0.08)	(−0.21 to −0.17)	(−0.23 to −0.19)	(−0.05 to −0.02)
Proportion attributable	0.47	0.04	0.96	0.54	0.35	0.36
	(0.43 to 0.50)	(−0.02 to 0.09)	(0.77 to 1.15)	(0.48 to 0.61)	(0.31 to 0.38)	(0.16 to 0.56)
Overall proportion mediated	0.44	−0.02	1.03	0.33	0.52	0.16
	(0.42 to 0.46)	(−0.05 to 0.01)	(0.87 to 1.19)	(0.30 to 0.37)	(0.50 to 0.55)	(0.04 to 0.28)
Overall proportion attributable to interaction	0.03	−0.07	0.09	−0.25	0.21	−0.39
	(−0.08 to 0. 14)	(−0.15 to 0.01)	(−0.11 to 0.28)	(−0.33 to −0.17)	(0.16 to 0.26)	(−0.77 to −0.009)
Overall proportion eliminated	0.43	−0.03	1.05	0.29	0.56	−0.03
	(0.41 to 0.46)	(−0.07 to 0.01)	(0.88 to 1.22)	(0.25 to 0.33)	(0.53 to 0.59)	(−0.28 to 0.22)

*Scenario 1: effect of low social class in both childhood and adulthood on (lower) physical capability, with partial mediation. Scenario 2: effect of social class in childhood on physical capability, with no mediation by adult social class. Scenario 3: effect of social class in childhood on physical capability, with complete mediation by adult social class. Scenario 4: effects of low social class in both childhood and adulthood on (lower) physical capability, with an interaction term that is in the opposite direction to the main effects of childhood and adult social class. Scenario 5: effects of low social class in both childhood and adulthood on (lower) physical capability, with an interaction in the same direction as the main effects of childhood and adult social class.

**Table 5.** Life course hypotheses selected by the structured approach within five simulated scenarios (*N* = 20 000) and the real-data example (*N* = 2122)

	Scenario 1*	Scenario 2*	Scenario 3*	Scenario 4*	Scenario 5*	Real-data example
First selected component of hypothesis	Accumulation	Early life critical period	Adult critical period	Accumulation	Accumulation	Accumulation
Second selected component of hypothesis	Adult critical period	Ever exposed	Always exposed	Adult critical period	Always exposed	Ever exposed
*P-*value for adding second component**	<0.001	0.45	0.70	<0.001	<0.001	0.48

*Scenario 1: effect of low social class in both childhood and adulthood on (lower) physical capability, with partial mediation. Scenario 2: effect of social class in childhood on physical capability, with no mediation by adult social class. Scenario 3: effect of social class in childhood on physical capability, with complete mediation by adult social class. Scenario 4: effects of low social class in both childhood and adulthood on (lower) physical capability, with an interaction term that is in the opposite direction to the main effects of childhood and adult social class. Scenario 5: effects of low social class in both childhood and adulthood on (lower) physical capability, with an interaction in the same direction as the main effects of childhood and adult social class.

**Low *P*-values indicate that adding the second hypothesis is supported, i.e. the second hypothesis makes an additional contribution to explaining the variance in the outcome. Since we only have two exposure time points, the value of adding a third hypothesis is not testable, as this would correspond to a fully saturated model.

In simulation 2 (effect of low social class in childhood on physical capability, with no mediation by adult social class), both forms of mediation analysis confirmed the presence of a direct effect of childhood social class with no mediation through adult social class (Tables and 4). The structured life course approach supported an early life critical period hypothesis (Table 5), which is compatible with this. Conversely, in scenario 3 (effect of low social class in childhood on physical capability, with complete mediation by adult social class), both sets of mediation analysis correctly suggested a strong indirect effect but no direct effect or interaction, and consistent with this the structured life course approach supported an adult critical period model.

In scenario 4 (interaction term in opposite direction to main effects of childhood and adult social class), the conventional mediation analysis (Table 3) identified this interaction. However, this is an example of how this method can be misleading if exposure-mediator interactions are present in the data but ignored in the analysis; in this scenario, the direct and indirect effects estimated in the whole sample are not appropriate because they ignore the interaction. When the interaction term is included in the conventional analysis, mediation can no longer be specified. The four-way decomposition analysis also identified the interaction, with a stronger mediated interaction term compared with the reference interaction term (Table 4). When the interaction goes in the opposite direction to the main effect, the proportions attributable to each component are not interpretable (interaction parameters have negative proportions). In this scenario, the structured life course approach supports accumulation and adult critical period hypotheses (Table 5), which implies a direct and indirect effect but no interaction parameters (Tables 1 and 2).

In scenario 5 (interaction term in the same direction as the main effects of childhood and adult social class), both mediation methods identify the presence of a direct and an indirect effect and an interaction (Tables 3 and 4), but, unlike in scenario 4, the proportions attributable to each term in the four-way decomposition are interpretable. Consistent with our expectations from Tables 1 and 2, the structured life course approach supports a combination of both accumulation (implies the presence of direct and indirect effects) and ever exposed (implies the presence of interaction terms) hypotheses.

### Real-data example results

Descriptive characteristics of participants included in the real-data example are shown in Appendix Table 1 (available as Supplementary Data at *IJE* online); as is common in cohort studies with long follow-up, women who remain actively engaged with the cohort tend to be of higher socioeconomic position than the full cohort at baseline.28 The ALSPAC data most closely mimic scenario 4, i.e. there is mediation but also an interaction such that there was a negative effect of low social class in childhood on physical capability in those who had high social class in adulthood, but no effect of low social class in childhood on physical capability in those who also had low social class in adulthood. As in the simulated dataset, both forms of mediation analysis identified a direct and indirect effect and the presence of the interaction (Tables 3 and 4), but the structured life course approach supported the accumulation model and did not select any of the hypotheses that suggest the presence of an interaction term (Table 5).

## Discussion

### Main findings

We have used theory and simulated datasets to describe the links between two forms of mediation analysis and the structured life course approach, showing which mediation and interaction parameters are present under each of a set of life course hypotheses. The relationship between the three methods was further exemplified using illustrative real data.

Our simulation studies and the real-data example have shown that the links between the methods are less clear when the main effects of the exposure/mediator and any statistical interaction between them are in opposite directions; in this case the mediation methods identified the presence of the interaction, but the structured life course approach supported the accumulation and adult critical period hypotheses, which suggests that only a direct and an indirect effect are present. However, it is important to note that the accumulation hypothesis, which suggests that in a mutually adjusted model the two exposures have equal magnitude effects on the outcome, is not explicitly tested within a mediation analysis framework.

### Limitations of our approach

In our examples, we have focused on the case of a binary exposure, binary mediator and continuous outcome. Whereas the conventional approaches to mediation analysis are simpler to implement with a continuous outcome,^9^ counterfactual-based methods in the presence of binary outcomes or mediators are well developed.^10^ The binary mediator necessitated the use of the difference method for estimating indirect effects.^13^ Binary exposures and mediators also have implications in terms of the life course hypotheses – for example, only people who are exposed (low SEP in early life) can contribute to the ‘upwards mobility’ hypothesis.

Since our simulations were intended to be illustrative rather than to assess the overall performance of the methods, we simulated only a single dataset for each of a limited number of scenarios. Alternative scenarios may result in different findings and conclusions. However, we have used a large sample size to minimize the influence of sampling variation. In some of our scenarios, the direct and indirect effects are in opposite directions, leading to negative attributable proportions. In this situation, MacKinnon recommends computing absolute percentages of mediated effects.^34^

### Results of real-data example

The results from our analysis of the real-data example were surprising; in previous literature it is more common to identify interactions between childhood and adult social class that are in the same direction as the main effects such that the worst outcomes are experienced by people who experience cumulative socioeconomic disadvantage.^35–37^ However, it is important to note that we intended these analyses to be illustrative of the links between mediation analysis and the structured life course approach, and therefore have not given full consideration in the analysis to potential sources of bias. For example, it is possible that other aspects of socioeconomic conditions and lifestyle in adulthood could act as intermediate confounders in this example.

### Questions asked by the approaches and parameters estimated

The questions asked by each of the approaches are summarized in Box 4. Mediation analysis using conventional regression analysis in the absence of an interaction allows us to examine the effect of the exposure on the outcome with adjustment only for confounders (total effect) and with adjustment for confounders and the later life exposure/mediator (direct effect). The indirect effect for the earlier measure with mediation via the later measure can be estimated using the product method (product of the paths exposure-mediator and mediator-outcome) or from the difference between the total and direct effects;^29^ a confidence interval can be calculated from this using path analysis,^38^ bootstrapping or other similar techniques.^39^ Total effects from conventional regression analysis are interpreted as the change in outcome associated with a one unit change in the exposure, adjusted for confounders. Direct effects are interpreted as the change in outcome per one unit higher exposure after adjustment for the mediator. In terms of interventions, this corresponds to the effect of altering the exposure while holding the mediator constant, i.e. in our example this would involve an intervention which shifted people's childhood social class from low to high, with all people having the same adult social class.

**Box 4.** Questions asked by each approach

Approach	Questions asked*
Mediation analysis using conventional regression approaches	To what extent is the effect of an early life exposure on an outcome explained by a mediator?
Interaction analysis using conventional regression approaches	Is there an interaction between an early life exposure and a mediator with respect to an outcome, and if so what is the effect of the early life exposure on the outcome within each stratum of the mediator?
Mediation and interaction analysis using four-way decomposition	To what extent do the four decomposition parameters contribute to the effect of an early life exposure on an outcome?
Structured life course approach	Which life course hypothesis best explains the relationship between the life course exposures and the outcome?

*Note that in our example, the mediator is a later life measure of the exposure variable, but in other applications the exposure and mediator could be different factors and could potentially have been measured at the same time, under the assumption of causal ordering of the two variables.

Once an interaction is included between the early life exposure and the mediator in conventional regression analyses, we obtain estimates of the effect of exposure on outcome separately for each stratum of the mediator. In a model including an interaction term, we cannot therefore explore mediation. This is a major limitation of conventional regression analyses, and a strong motivation for using one of the more recently developed methods for decomposing the total exposure effect on the outcome.^12^

Counterfactual theory has facilitated the definition and estimation of the direct [CDE(m)] effect in the presence of measured intermediate confounders and indirect effects even in the presence of non-linearity, and exposure-mediator interactions.^10^^,^^11^^,^^40^ Decomposition analyses, such as the approach used here,^12^ are one application of these methods based on counterfactual theory, and allow the contribution of mediation and exposure-mediator interaction to be estimated simultaneously. One of the quantities of interest that this approach defines and estimates is the relative contribution of each of the decomposition parameters. For these estimates to be meaningful, the direction of effects needs to be the same (i.e. all positive or all negative). In our real-data example, the direct and indirect effect are negative whereas the interaction terms are positive. This means that the proportions attributable to each of the estimated parameters in the model are not sensible or interpretable.^12^(12)

The structured life course approach does not consider mediation per se. Rather, it identifies the life course hypothesis (or hypotheses) that best fits the data in terms of the relationship between life course exposures and an outcome. With the lasso approach, the alternative hypotheses are encoded by a series of separate statistical models (e.g. for an early life critical period hypothesis, the statistical model would include only an intercept and a coefficient for early life exposure), and the procedure selects the hypothesis/hypotheses that explains the most variance in the outcome. We have shown which mediation parameters, both from conventional regression analyses and from the four-way decomposition analysis, would be present under each of the commonly used life course hypotheses.

### Assumptions of the approaches

All approaches considered here share a common set of assumptions: that there is no unmeasured confounding between the exposure and the mediator, the exposure and the outcome or the mediator and the outcome, and no measurement error, particularly in the mediator.^3–5^(3–5) Thus although mediation methods based on counterfactual theory have enabled the relaxation of certain model assumptions compared with conventional regression methods [e.g. permitting non-linear relationships, and estimation of mediation parameters in the presence of exposure-mediator interactions and the CDE(m) in the presence of measured intermediate confounders], they still rely on these same fundamental assumptions. In some cases, it may be possible to apply methods to life course studies that are robust to confounding, for example Mendelian randomization or other instrumental variable approaches that can estimate unbiased effects even in the presence of unmeasured confounding,^41^^,^^42^ but this relies on the availability of suitable instruments for both the exposure and mediator. Furthermore, instrumental variable approaches have their own assumptions (some of which are not possible or easy to test) and are also subject to misclassification bias.^43^ Sensitivity analyses to the implications of unmeasured confounding and measurement error are recommended,^11^ and in some cases it may be possible to apply methods for correction of misclassification bias. For example, if sufficient replicate data are available, it may be possible to use methods such as regression calibration. The advancement of methods to evaluate and potentially deal with misclassification bias is an area ripe for methodological development.

### Which method when?

Within life course epidemiology, our ultimate goal is generally to understand the potential impact of interventions targeted at different stages of the life course. Although addressing different questions, mediation analysis and the structured life course approach are two alternative approaches for the analysis of life course data. Clarifying the relationships between them is important in terms of understanding how the methods relate to one another, and to aid comparisons of results from different studies that have used one of the two methods to address the same question.

Typically, and in our example analyses, the structured life course approach has been used when the exposure and mediator are measures of the same construct from different points of the life course. Careful thought would be needed as to the interpretation and therefore relevance of each life course hypothesis if using this approach for multiple different exposures.

In addition to the subtleties of the different questions asked by each approach, various practical considerations may be important in choosing between the methods. For example, conventional mediation approaches (including within a structural equation modelling framework) cannot incorporate non-linear relationships or non-linear models, whereas counterfactual-based approaches can.^44^ The number of measurements (i.e. repeated measures of an exposure, as in this example, or an exposure plus repeated measures of a mediator) is also an important consideration. The structured life course approach readily deals with three or more life course exposures. Structural equation models can incorporate repeated measures data, particularly if repeated measures of a mediator are captured by latent growth models, but require strong parametric assumptions to do so.^44^ Recent papers have outlined possible approaches to extending counterfactual-based mediation methods for repeated measures,^44–47^ but these remain complex and challenging.

Tackling a given research question using both mediation analysis (choosing between the conventional approach and a counterfactual approach, depending on whether relaxing the modelling assumptions made in the conventional approach is necessary within a specific example) and a structured life course approach offers a complementary set of information, and in many cases it may therefore be advisable to use both approaches in order to provide a fuller understanding of the life course relationships under study.

### Conclusion

Life course epidemiology seeks to understand how exposures across the life course come together to influence health. Many life course questions can be framed in terms of mediation and/or interaction. Newer mediation methods based on counterfactual theory have advantages over conventional mediation analysis since they enable, for example, exposure-mediator interactions, appropriate treatment of intermediate confounders, and non-linear relationships. The structured life course approach is an alternative to mediation analysis, which selects the life course hypothesis or hypotheses that best fit the observed data. We have shown that mediation analysis and the structured life course approach can, in most scenarios, be considered as intrinsically linked, since each life course hypothesis suggests the presence of a specific set of mediation and/or interaction terms.

## Funding

This work was supported by a grant from the UK Economic and Social Research Council (ES/M010317/1). L.D.H., C.M.W. and A.F. are supported by fellowships from the UK Medical Research Council (MR/M020894/1, MR/J011932/1 and MR/M009351/1, respectively). Research reported in this publication was supported by the National Institute on Aging of the National Institutes of Health under Award No. R01AG048835. The content is solely the responsibility of the authors and does not necessarily represent the official views of the National Institutes of Health. L.D.H., C.M.W., E.A., D.A.L., K.T. and A.F. work in a unit that receives funding from the University of Bristol and the UK Medical Research Council (MC_UU_12013/5 and MC_UU_12013/9). R.C. and R.H. are supported by the UK Medical Research Council (Programme codes MC_UU_12019/4 and MC_UU_12019/2, respectively). B.G. was funded by a Wellcome Trust Fellowship (No. 089979). The UK Medical Research Council, the Wellcome Trust (grant ref: 092731) and the University of Bristol provided core support for the Avon Longitudinal Study of Parents and their Children. The ALSPAC mother's study (data for which were used in our example) is funded by the British Heart Foundation (SP/07/008/24066), Medical Research Council (G1001357) and Wellcome Trust (WT092830M).

## Supplementary Material

Supplementary Data

Supplementary Data

## Appendix: Derivation of relationship between decomposition analyses and life course models

VanderWeele(12) showed that, under certain conditions, when the exposure and mediator are both binary variables, the 4-way decomposition in **Box 1** estimates the following components, which are reproduced here using counterfactual notation:

**Table:**

CDE(0)	*Y*_10_ – *Y*_00,_
INT_ref_	(*Y*_11_ – *Y*_10_ – *Y*_01_ + *Y*_00_) *M*_0,_
INT_med_	(*Y*_11_ – *Y*_10_ – *Y*_01_ + *Y*_00_) (*M*_1_ – *M*_0_),
PIE	(*Y*_01_ – *Y*_00_) (*M*_1_ – *M*_0_),

*Y_xm_* is the value of the outcome (Y) if the exposure (X) were set to *x* and mediator (M) were set to *m*, and *M_x_* is the value of the mediator if the exposure were set to *x*.

The seven life course models in **Box 2** can be summarized by how the potential outcome differs between levels of the exposure and mediator:

**Table:**

*Early life critical period*	*Y*_00_ = *Y*_01_ ≠ *Y*_10_ = *Y*_11_
*Adult critical period*	*Y*_00_ = *Y*_10_ ≠ *Y*_01_ = *Y*_11_
*Accumulation*	*Y*_01_ = *Y*_10_, *Y*_11_ – *Y*_10_ = *Y*_01_ – *Y*_00_ ≠ 0
*Increasing social class*	*Y*_10_ ≠ *Y*_00_ = *Y*_01_ = *Y*_11_
*Decreasing social class*	*Y*_01_ ≠ *Y*_00_ = *Y*_10_ = *Y*_11_
*Always exposed*	*Y*_00_ = *Y*_01_ = *Y*_10_ ≠ *Y*_11_
*Ever exposed*	*Y*_00_ ≠ *Y*_01_ = *Y*_10_ = *Y*_11_

Using these equalities and inequalities, we can quickly see which of the components of the 4-way decomposition are non-zero.

The **controlled direct effect** will be non-zero when *Y*_10_ – *Y*_00_ ≠ 0. Therefore the *early life critical period*, *accumulation*, *increasing social class* and *ever exposed* models will show a controlled direct effect.

The **pure indirect effect** can only be non-zero if *Y*_01_ – *Y*_00_ ≠ 0, which happens under the *adult critical adulthood*, *accumulation*, *decreasing social class* and *ever exposed* models. Therefore a pure indirect effect will show in these models provided that *M*_1_ – *M*_0_ ≠ 0, i.e. there is an association between exposure and mediator.

The **reference interaction** will be non-zero if *Y*_11_ – *Y*_10_ – *Y*_01_ + *Y*_00_ ≠ 0, under the reasonable assumption that *M*_0_ ≠ 0. Therefore the *increasing social class*, *decreasing social class*, *always exposed* and *ever exposed* models will show a reference interaction.

The **mediated interaction** can only be non-zero if *Y*_11_ – *Y*_10_ – *Y*_01_ + *Y*_00_ ≠ 0, which occurs under the *increasing social class*, *decreasing social class*, *always exposed* and *ever exposed* models. Therefore a mediated interaction will show in these models provided that *M*_1_ – *M*_0_ ≠ 0.

The **total indirect effect** is TIE = PIE + INT_med_. In general, this will be non-zero if PIE and/or INT_med_ are non-zero. Therefore a total indirect effect will show in the *critical adulthood*, *accumulation*, *increasing social class* and *always exposed* models, provided that *M*_1_ – *M*_0_ ≠ 0. However, in the decreasing social class and ever exposed models, we have *Y*_11_ – *Y*_10_ = 0 and hence PIE = –INT_med_, so a total indirect effect will not show in these models in spite of having non-zero PIE and non-zero INT_med_.

To consider the conventional direct and indirect effects in mediation analysis, we switch from counterfactuals to regression models. Suppose then that the outcome can be modelled by the linear model with interaction:

Y=α+βX+γM+δXM+ε,

where E(*ε*) = 0 and *ε* is independent of the binary *X* and *M*. The seven life course models in **Box 2** can be summarized by the relationships of the regression parameters *β*, *γ* and *δ*:

**Table:**

*Early life critical period*	*β* ≠ 0, *γ* = 0, *δ* = 0
*Adult critical period*	*β* = 0, *γ* ≠ 0, *δ* = 0
*Accumulation*	*β* = *γ* ≠ 0, *δ* = 0
*Increasing social class*	*β* = -*δ* ≠ 0, *γ* = 0
*Decreasing social class*	*β* = 0, *γ* = -*δ* ≠ 0
*Always exposed*	*β* = 0, *γ* = 0, *δ* ≠ 0
*Ever exposed*	*β* = *γ* = -*δ* ≠ 0

The conventional **Direct Effect** is the regression coefficient of *Y* on *X*, adjusted for *M*. This can be calculated using a standard formula:

$$\left(\text{var}\left(M\right)\;\text{cov}\left(X,Y\right)\;–\;\text{cov}\left(X,M\right)\;\text{cov}\left(M,Y\right)\right)\;/\;(\text{var}\left(M\right)\;\text{var}\left(X\right)\;–\;\text{cov}{\left(X,M\right)}^{2}).$$

The covariances with the outcome are

$$\begin{array}{ccc} \text{cov}\left(X,Y\right) & = & \text{cov}\left(X,\alpha +\beta X+\gamma M+\delta XM+\varepsilon \right)\\ & = & \text{cov}\left(X,\alpha \right)+\text{cov}\left(X,\beta X\right)+\text{cov}(X,\gamma M)\\ & + & \text{cov}\left(X,\delta XM\right)+\text{cov}\left(X,\varepsilon \right)\\ & = & \beta \text{var}\left(X\right)+\gamma \text{cov}\left(X,M\right)+\delta \text{cov}\left(X,XM\right) \end{array}$$

and

$$\begin{array}{ccc} \text{cov}\left(M,Y\right) & = & \text{cov}\left(M,\alpha +\beta X+\gamma M+\delta XM+\varepsilon \right)\\ & = & \text{cov}\left(M,\alpha \right)+\text{cov}\left(M,\beta X\right)+\text{cov}\left(M,\gamma M\right)\\ & + & \text{cov}\left(M,\delta XM\right)+\text{cov}\left(M,\varepsilon \right)\\ & = & \beta \text{cov}\left(X,M\right)+\gamma \;var\left(M\right)+\delta \text{cov}(M,XM). \end{array}$$

Therefore the numerator in the direct effect formula is *β* var(*M*) var(*X*) + *γ* var(*M*) cov(*X*,*M*) + *δ* var(*M*) cov(*X*,*XM*) – *β* cov(*X*,*M*)^2^ – *γ* var(*M*) cov(*X*,*M*) – *δ* cov(*X*,*M*) cov(*M*,*XM*). The direct effect is consequently

$$\beta +\delta \left(\text{var}\left(M\right)\text{cov}\left(X,XM\right)\;–\;\text{cov}\left(X,M\right)\text{cov}\left(M,XM\right)\right)\;/\;(\text{var}\left(M\right)\;\text{var}\left(X\right)\;–\;\text{cov}{\left(X,M\right)}^{2}).$$

The fact that *X* and *M* are binary means that var(*X*) = E(*X*)(1–E(*X*)) and var(*M*) = E(*M*)(1–E(*M*)). Furthermore E(*XM*) = E(*X^2^M*) = E(*XM^2^*), so that cov(*X*,*XM*) = E(*X*^2^*M*)–E(*X*)E(*XM*) = (1–E(*X*))E(*XM*) and cov(M,*XM*) = E(*XM*^2^)–E(*M*)E(*XM*) = (1–E(*M*))E(*XM*). To see that the coefficient for *δ* in the direct effect is non-zero, note that substituting the above expressions into var(*M*) cov(*X*,*XM*) – cov(*X*,*M*) cov(*M*,*XM*) gives (1–E(*M*))E(*XM*)(E(*M*)–E(*XM*)), which can only be zero if E(M) = 1 or E(*XM*) = 0, or if E(*XM*) = E(*M*). These conditions cannot happen if all combinations of *X* and *M* occur with non-zero probability. It is possible that the two terms in the direct effect will cancel each other by chance. This is a disadvantage that is not present in the 4 way decomposition. Ignoring this possibility, it is easier to identify the models in which the direct effect is zero. This will occur if both *β* = 0 and *δ* = 0. The only model for which this is the case, and there is no direct effect, is the *adult critical period* model.

The conventional **Indirect Effect** is the product of the regression coefficient of *Y* on *M*, adjusted for *X*, and the regression coefficient of *M* on *X*. The adjusted regression coefficient is

$$\left(\text{var}\left(X\right)\;\text{cov}\left(M,Y\right)\;–\;\text{cov}\left(X,M\right)\;\text{cov}\left(X,Y\right)\right)\;/\;(\text{var}\left(M\right)\;\text{var}\left(X\right)\;–\;\text{cov}{\left(X,M\right)}^{2}),$$

with numerator *β* var(*X*) cov(*X*,*M*) + *γ* var(*X*) var(*M*) + *δ* var(*X*) cov(*M*,*XM*) – *β* var(*X*) cov(*X*,*M*) – *γ* cov(*X*,*M*)^2^ – *δ* cov(*X*,*M*) cov(*X*,*XM*). The regression coefficient of *M* on *X* is cov(*X*,*M*) / var(*X*), and the indirect effect is therefore

$$\left(\text{cov}\left(X,M\right)\;/\;\text{var}\left(X\right)\right)\;\left(\gamma +\delta \left(\text{var}\left(X\right)\text{cov}\left(M,XM\right)\;–\;\text{cov}\left(X,M\right)\text{cov}\left(X,XM\right)\right)\;/\;\left(\text{var}\left(M\right)\;\text{var}\left(X\right)\;–\;\text{cov}{\left(X,M\right)}^{2}\right)\right)$$

The coefficient for *δ* is (1–E(*X*))E(*XM*)(E(*X*)–E(*XM*)), which once again cannot be zero if all combinations of *X* and *M* occur with non-zero probability. The indirect effect will always be zero unless cov(*X*,*M*) ≠ 0, i.e. there is an association between exposure and mediator. Again ignoring chance cancellation, it is simpler to show the models in which the indirect effect is zero. This is only certain to occur when *γ* = 0 and *δ*= 0. Only in the *early critical period* model will this occur and there be no Indirect Effect.

Some life course models can be thought of as a combination of simpler models. For instance a ‘sensitive period’ hypothesis can be thought of as a combination of a critical period and accumulation models. Should such a model be identified, all effects that are non-zero in the component models could be considered to be non-zero in the identified compound model. There is the possibility that the effects of the separate component models may cancel each other out by chance.

## References

[1] Kuh D Ben-Schlomo Y Lynch J Hallqvist J Power C Life course epidemiology. J Epidemiol Community Health 2003;57**:** 778–83. 14573579 10.1136/jech.57.10.778 PMC1732305

[2] Baron RM Kenny DA The moderator-mediator variable distinction in social psychological research: conceptual, strategic, and statistical considerations. J Pers Soc Psychol 1986;51**:** 1173–82. 3806354 10.1037//0022-3514.51.6.1173

[3] Blakely T McKenzie S Carter K Misclassification of the mediator matters when estimating indirect effects. J Epidemiol Community Health 2013;67**:** 458–66. 23386673 10.1136/jech-2012-201813

[4] Valeri L Vanderweele TJ The estimation of direct and indirect causal effects in the presence of misclassified binary mediator. Biostatistics 2014;15**:** 498–512. 24671909 10.1093/biostatistics/kxu007 PMC4059465

[5] VanderWeele TJ Valeri L Ogburn EL The role of measurement error and misclassification in mediation analysis: mediation and measurement error. Epidemiology 2012;23**:** 561–64. 22659547 10.1097/EDE.0b013e318258f5e4 PMC3367328

[6] Richiardi L Bellocco R Zugna D Mediation analysis in epidemiology: methods, interpretation and bias. Int J Epidemiol 2013;42**:** 1511–19. 24019424 10.1093/ije/dyt127

[7] Cole SR Platt RW Schisterman EF Chu H Westreich D Richardson D et al. Illustrating bias due to conditioning on a collider. Int J Epidemiol 2010;39**:** 417–20. 19926667 10.1093/ije/dyp334 PMC2846442

[8] Greenland S Quantifying biases in causal models: classical confounding vs collider-stratification bias. Epidemiology 2003;14**:** 300–06. 12859030

[9] Kenny DA Mediation, 22nd May 2016. http://davidakenny.net/cm/mediate.htm (29 August 2016, date last accessed).

[10] VanDerWeele T Mediation analysis: a practitioner's guide. Annu Rev Public Health 2016;37**:** 17–32. 26653405 10.1146/annurev-publhealth-032315-021402

[11] VanDerWeele T Explanation in Causal Inference: Methods for Mediation and Interaction. New York, NY: Oxford University Press, 2015.

[12] VanderWeele TJ A unification of mediation and interaction: a 4-way decomposition. Epidemiology 2014;25**:** 749–61. 25000145 10.1097/EDE.0000000000000121 PMC4220271

[13] Vanderweele TJ Vansteelandt S Robins JM Effect decomposition in the presence of an exposure-induced mediator-outcome confounder. Epidemiology 2014;25**:** 300–06. 24487213 10.1097/EDE.0000000000000034 PMC4214081

[14] Barker DJP Osmond C Forsen TJ Kajantie E Eriksson JG Trajectories of growth among children who have coronary events as adults. N Engl J Med 2005;353**:** 1802–09. 16251536 10.1056/NEJMoa044160

[15] VanderWeele TJ On the distinction between interaction and effect modification. Epidemiology 2009;20**:** 863–71. 19806059 10.1097/EDE.0b013e3181ba333c

[16] Naimi AI Kaufman JS MacLehose RF Mediation misgivings: ambiguous clinical and public health interpretations of natural direct and indirect effects. Int J Epidemiol 2014;43**:** 1656–61. 24860122 10.1093/ije/dyu107

[17] Ben-Schlomo Y Kuh D A life course approach to chronic disease epidemiology: conceptual models, empirical challenges and interdisciplinary perspectives. Int J Epidemiol 2002;31**:** 285–93. 11980781

[18] Smith AD Heron J Mishra G Gilthorpe MS Ben-Shlomo Y Tilling K Model selection of the effect of binary exposures over the life course. Epidemiology 2015;26**:** 719–26. 26172863 10.1097/EDE.0000000000000348 PMC4521897

[19] Mishra G Nitsch D Black S De SB Kuh D Hardy R A structured approach to modelling the effects of binary exposure variables over the life course. Int J Epidemiol 2009;38**:** 528–37. 19028777 10.1093/ije/dyn229 PMC2663717

[20] Kuh D Karunananthan S Bergman H Cooper R A life-course approach to healthy ageing: maintaining physical capability. Proc Nutr Soc 2014;73**:** 237–48. 24456831 10.1017/S0029665113003923 PMC3981474

[21] Lara J Cooper R Nissan J et al. A proposed panel of biomarkers of healthy ageing. BMC Med 2015;13**:** 222. 26373927 10.1186/s12916-015-0470-9 PMC4572626

[22] Cooper R Strand BH Hardy R Patel KV Kuh D Physical capability in mid-life and survival over 13 years of follow-up: British birth cohort study. BMJ 2014;348**:** g2219. 24787359 10.1136/bmj.g2219 PMC4004787

[23] Birnie K Cooper R Martin RM et al. Childhood socioeconomic position and objectively measured physical capability levels in adulthood: a systematic review and meta-analysis. PloS One 2011;6**:** e15564. 21297868 10.1371/journal.pone.0015564 PMC3027621

[24] Murray ET Ben-Shlomo Y Tilling K et al. Area deprivation across the life course and physical capability in midlife: findings from the 1946 British Birth cohort. Am J Epidemiol 2013;178**:** 441–50. 23788665 10.1093/aje/kwt003 PMC3727343

[25] Hurst L Stafford M Cooper R Hardy R Richards M Kuh D Lifetime socioeconomic inequalities in physical and cognitive aging. Am J Public Health 2013;103**:** 1641–48. 23865666 10.2105/AJPH.2013.301240 PMC3780680

[26] Strand BH Cooper R Hardy R Kuh D Guralnik J Lifelong socioeconomic position and physical performance in midlife: results from the British 1946 birth cohort. Eur J Epidemiol 2011;26**:** 475–83. 21416275 10.1007/s10654-011-9562-9 PMC3246593

[27] Birnie K Martin RM Gallacher J et al. Socio-economic disadvantage from childhood to adulthood and locomotor function in old age: a lifecourse analysis of the Boyd Orr and Caerphilly prospective studies. J Epidemiol Community Health 2011;65**:** 1014–23. 20644236 10.1136/jech.2009.103648 PMC3381706

[28] Fraser A Macdonald-Wallis C Tilling K et al. Cohort Profile: The Avon Longitudinal Study of Parents and Children: ALSPAC mothers cohort. Int J Epidemiol 2013;42**:** 97–110. 22507742 10.1093/ije/dys066 PMC3600619

[29] Jiang Z VanderWeele TJ When is the difference method conservative for assessing mediation?. Am J Epidemiol 2015;182**:** 105–08. 25944885 10.1093/aje/kwv059 PMC4493982

[30] Ikram MA VanderWeele TJ A proposed clinical and biological interpretation of mediated interaction. Eur J Epidemiol 2015;30**:** 1115–18. 26438385 10.1007/s10654-015-0087-5 PMC4636997

[31] Lockhart R Taylor J Tibshirani RJ Tibshirani R A significance test for the lasso. Ann Stat 2014;42**:** 413–68. 25574062 10.1214/13-AOS1175 PMC4285373

[32] StataCorp. Stata Statistical Software: Release 13. College Station, TX: StataCorp, 2013.

[33] R Core Development Team. R: A Language and Environment for Statistical Computing. 3.0.1 edn Vienna: R Foundation for Statistical Computing, 2015.

[34] MacKinnon DP Introduction to Statistical Mediation Analysis. New York, NY: Taylor and Francis, 2008.

[35] Lyu J Burr JA Socioeconomic status across the life course and cognitive function among older adults: an examination of the latency, pathways, and accumulation hypotheses. J Aging Health 2016;28**:** 40–67. 26006338 10.1177/0898264315585504

[36] Seabrook JA Avison WR Socioeconomic status and cumulative disadvantage processes across the life course: implications for health outcomes. Can Rev Sociol 2012;49**:** 50–68. 22586837 10.1111/j.1755-618x.2011.01280.x

[37] Stringhini S Batty GD Bovet P et al. Association of lifecourse socioeconomic status with chronic inflammation and type 2 diabetes risk: the Whitehall II prospective cohort study. PLoS Med 2013;10**:** e1001479. 23843750 10.1371/journal.pmed.1001479 PMC3699448

[38] Wright S The method of path coefficients. Ann Math Stat 1934;5**:** 161–215.

[39] Boos DD Osborne JA Assessing variability of complex descriptive statistics in monte carlo studies using resampling methods. Int Stat Rev 2015;83**:** 228–38. 26345317 10.1111/insr.12087 PMC4556306

[40] Valeri L Vanderweele TJ Mediation analysis allowing for exposure-mediator interactions and causal interpretation: theoretical assumptions and implementation with SAS and SPSS macros. Psychol Methods 2013;18**:** 137–50. 23379553 10.1037/a0031034 PMC3659198

[41] Burgess S Daniel RM Butterworth AS Thompson SG Network Mendelian randomization: using genetic variants as instrumental variables to investigate mediation in causal pathways. Int J Epidemiol 2015;44**:** 484–95. 25150977 10.1093/ije/dyu176 PMC4469795

[42] Relton CL Davey Smith G Two-step epigenetic Mendelian randomization: a strategy for establishing the causal role of epigenetic processes in pathways to disease. Int J Epidemiol 2012;41**:** 161–76. 22422451 10.1093/ije/dyr233 PMC3304531

[43] Pierce BL VanderWeele TJ The effect of non-differential measurement error on bias, precision and power in Mendelian randomization studies. Int J Epidemiol 2012;41**:** 1383–93. 23045203 10.1093/ije/dys141

[44] De Stavola BL Daniel RM Ploubidis GB Micali N Mediation analysis with intermediate confounding: structural equation modeling viewed through the causal inference lens. Am J Epidemiol 2015;181**:** 64–80. 25504026 10.1093/aje/kwu239 PMC4383385

[45] Daniel RM De Stavola BL Cousens SN Vansteelandt S Causal mediation analysis with multiple mediators. Biometrics 2015;71**:** 1–14. 25351114 10.1111/biom.12248 PMC4402024

[46] VanDerWeele T Vansteelandt S Mediation analysis with multiple mediators. Epidemiol Methods 2013;2**:** 95–115. 10.1515/em-2012-0010 PMC4287269 25580377

[47] Bind MA Vanderweele TJ Coull BA Schwartz JD Causal mediation analysis for longitudinal data with exogenous exposure. Biostatistics 2016;17**:** 122–34. 26272993 10.1093/biostatistics/kxv029 PMC4731412

