# Supplementary material for: Relationship between mediation analysis and the structured life course approach
Source: Int J Epidemiol. 2016 Oct 6;45(4):1280–94. doi: 10.1093/ije/dyw254 (PMC5841634; doi:10.1093/ije/dyw254)
Supplement: Supplementary Data [file dyw254_supplementary_data.zip › ije-2015-10-1447-File002.docx]

**Online only supplementary material for:**

**Relationship between mediation/interaction analysis and the structured life course approach**

Laura D Howe^1,2^*, Andrew D Smith^1,2^, Corrie Macdonald-Wallis^1,2^, Emma L Anderson^1,2^, Bruna Galobardes^2^, Debbie A Lawlor^1,2^, Yoav Ben-Shlomo^2^, Rebecca Hardy^3^, Rachel Cooper^3^, Kate Tilling^1,2^, Abigail Fraser^1,2^

1 – MRC Integrative Epidemiology Unit at the University of Bristol, Bristol, UK

2 – School of Social and Community Medicine, University of Bristol, Bristol, UK

3 – MRC Unit for Lifelong Health and Ageing at UCL, London, UK

* Corresponding author. Oakfield House, Oakfield Grove, Bristol BS8 2BN, UK. [laura.howe@bristol.ac.uk](mailto:laura.howe@bristol.ac.uk). +44 (0)117 3310134

**Details of occupational social class measures:**

Occupational social class in childhood was based on self-reported paternal occupation, coded as classes I (professional/managerial) to V (unskilled manual workers), using the 1991 British Office of Population and Census Statistics (OPCS) classification. Women’s occupational social class in adulthood (at entry into the study, mean age 30.2 years), was based on self-reported own and/or partner’s occupation (the highest of the two), according to the above categories. For simplicity in our illustrative example, occupational social class was dichotomised as low (unskilled, partly skilled, skilled manual and skilled non-manual) and high (managerial and technical, and professional), with this definition being used instead of the more conventional manual/non-manual dichotomisation because of the low number of women with manual social class in this cohort.

**Details of physical capability measures and score derivation:**

Hand grip strength was assessed using a Jamar handgrip dynamomenter. Two values were recorded for each hand and the highest value was used in analyses. Grip strength was adjusted for height by diving by height. Chair rise time was measured with a stopwatch as the time taken to rise from a sitting to a standing position with straight back and legs and then to sit down again 10 complete times as fast as possible. Standing balance was assessed as the time, up to a maximum of 30 seconds that a one-legged stance could be maintained with eyes closed. Participants were also timed walking at their normal pace over 3 metres. To create an overall score of physical capability and hence to simplify analyses, each of the four individual measures were rescaled so that values lay between 0 and 1, with larger values indicating greater physical capability, i.e. reversing the scores for chair rise and walking times. Extreme values for each measure were assigned the value of the 99th centile, as in previous studies. If a study participant was unable to perform the test for health reasons they were assigned a value of 0. The measures were then added to create a score ranging from 0 (low performance) to 4 (high performance). Women’s age at assessment was also recorded, and the physical capability score was regressed on age; the residuals of this regression were used as an age-adjusted measure of physical capability as the outcome in these analyses. Women with complete data on all key variables (N=2,122) were included in our analyses.

**Stata code for generating simulated datasets:**

**Simulation 1

clear all

set obs 20000

gen csocclass=invnorm(uniform())

gen asocclass=csocclass*0.25+0.5+invnorm(uniform())*0.25

gen csoc=csocclass<=0

gen asoc=asocclass<=0.3

gen outcome=csoc*0.25+asoc*0.5+invnorm(uniform())*0.5

gen newoutcome=(outcome+1.545879)

replace newoutcome=0 if newoutcome<=0.001

replace newoutcome = 4-newoutcome

**Simulation 2

clear all

set obs 20000

gen csocclass=invnorm(uniform())

gen asocclass=csocclass*0.25+0.5+invnorm(uniform())*0.25

gen csoc=csocclass<=0

gen asoc=asocclass<=0.3

gen outcome=csoc*0.25+invnorm(uniform())*0.5

gen newoutcome=(outcome+1.78)

replace newoutcome=0 if newoutcome<=0.001

replace newoutcome = 4-newoutcome

**Simulation 3

clear all

set obs 20000

gen csocclass=invnorm(uniform())

gen asocclass=csocclass*0.25+0.5+invnorm(uniform())*0.25

gen csoc=csocclass<=0

gen asoc=asocclass<=0.3

gen outcome=asoc*0.25+invnorm(uniform())*0.5

gen newoutcome=(outcome+1.78)

replace newoutcome=0 if newoutcome<=0.001

replace newoutcome = 4-newoutcome

**Simulation 4

clear all

set obs 20000

gen csocclass=invnorm(uniform())

gen asocclass=csocclass*0.25+0.5+invnorm(uniform())*0.25

gen csoc=csocclass<=0

gen asoc=asocclass<=0.3

gen outcome=csoc*0.25+asoc*0.5+invnorm(uniform())*0.5

gen newoutcome=(outcome+1.545879)

replace newoutcome=0 if newoutcome<=0.001

replace newoutcome = 4-newoutcome

replace newoutcome = newoutcome+0.2+invnorm(uniform()) if csoc==1 & asoc==1

**Simulation 5

clear all

set obs 20000

gen csocclass=invnorm(uniform())

gen asocclass=csocclass*0.25+0.5+invnorm(uniform())*0.25

gen csoc=csocclass<=0

gen asoc=asocclass<=0.3

gen outcome=csoc*0.25+asoc*0.5+invnorm(uniform())*0.5

gen newoutcome=(outcome+1.545879)

replace newoutcome=0 if newoutcome<=0.001

replace newoutcome = 4-newoutcome

replace newoutcome = newoutcome-0.3+invnorm(uniform()) if csoc==1 & asoc==1

**Stata code used for four-way decomposition analysis, shown with a covariate (‘covariate’) for illustrative purposes:**

local yvar = "res"

local mvar = "adult_low"

local avar = "early_low"

* Only one confounder currently included but more could be added

local cvar = "covariate"

local m = 0

local c = 0

gen am_int = `avar'*`mvar'

regress `yvar' `avar' `mvar' am_int `cvar'

matrix b_y = e(b)

matrix V_y = e(V)

logit `mvar' `avar' `cvar'

matrix b_m = e(b)

matrix V_m = e(V)

local theta_0 = b_y[1,5]

local theta_1 = b_y[1,1]

local theta_2 = b_y[1,2]

local theta_3 = b_y[1,3]

local theta_4 = b_y[1,4]

local beta_0 = b_m[1,3]

local beta_1 = b_m[1,1]

local beta_2 = b_m[1,2]

matrix theta_beta = (`theta_0', `theta_1', `theta_2', `theta_3', `theta_4', `beta_0', `beta_1', `beta_2')

matrix var_theta_beta = (V_y[5,5], V_y[1,5], V_y[2,5], V_y[3,5], V_y[4,5], 0, 0, 0 \ ///

V_y[1,5], V_y[1,1], V_y[1,2], V_y[1,3], V_y[1,4], 0, 0, 0 \ ///

V_y[2,5], V_y[1,2], V_y[2,2], V_y[2,3], V_y[2,4], 0, 0, 0 \ ///

V_y[3,5], V_y[1,3], V_y[2,3], V_y[3,3], V_y[3,4], 0, 0, 0 \ ///

V_y[4,5], V_y[1,4], V_y[2,4], V_y[3,4], V_y[4,4], 0, 0, 0 \ ///

0, 0, 0, 0, 0, V_m[3,3], V_m[1,3], V_m[2,3] \ ///

0, 0, 0, 0, 0, V_m[1,3], V_m[1,1], V_m[1,2] \ ///

0, 0, 0, 0, 0, V_m[2,3], V_m[1,2], V_m[2,2])

* Functions CDE, INTref, INTmed, PIE, TE and proportions:

local cde = `theta_1' + `theta_3'*`m'

local int_ref = `theta_3'*((exp(`beta_0' + `beta_2'*`c')/(1 + exp(`beta_0' + `beta_2'*`c'))) - `m')

local int_med = `theta_3'*((exp(`beta_0' + `beta_1' + `beta_2'*`c')/(1 + exp(`beta_0' + `beta_1' + `beta_2'*`c'))) - (exp(`beta_0' + `beta_2'*`c')/(1 + exp(`beta_0' + `beta_2'*`c'))))

local pie = `theta_2'*((exp(`beta_0' + `beta_1' + `beta_2'*`c')/(1 + exp(`beta_0' + `beta_1' + `beta_2'*`c'))) - (exp(`beta_0' + `beta_2'*`c')/(1 + exp(`beta_0' + `beta_2'*`c'))))

local te = `cde' + `int_ref' + `int_med' + `pie'

local prop_cde = `cde'/`te'

local prop_int_ref = `int_ref'/`te'

local prop_int_med = `int_med'/`te'

local prop_pie = `pie'/`te'

local overall_prop_med = (`pie' + `int_med')/`te'

local overall_prop_attr_int = (`int_ref' + `int_med')/`te'

local overall_prop_elim = (`int_ref' + `int_med' + `pie')/`te'

matrix f_theta_beta = (`cde' \ `int_ref' \ `int_med' \ `pie' \ `te' \ `prop_cde' \ `prop_int_ref' \ `prop_int_med' \ `prop_pie' \ `overall_prop_med' \ `overall_prop_attr_int' \ `overall_prop_elim')

local d_cde_d_theta_0 = 0

local d_cde_d_theta_1 = 1

local d_cde_d_theta_2 = 0

local d_cde_d_theta_3 = `m'

local d_cde_d_theta_4 = 0

local d_cde_d_beta_0 = 0

local d_cde_d_beta_1 = 0

local d_cde_d_beta_2 = 0

local d_int_ref_d_theta_0 = 0

local d_int_ref_d_theta_1 = 0

local d_int_ref_d_theta_2 = 0

local d_int_ref_d_theta_3 = (exp(`beta_0' + `beta_2'*`c')/(1 + exp(`beta_0' + `beta_2'*`c'))) - `m'

local d_int_ref_d_theta_4 = 0

local d_int_ref_d_beta_0 = `theta_3'*(exp(`beta_0' + `beta_2'*`c')/((1+exp(`beta_0' + `beta_2'*`c'))^2))

local d_int_ref_d_beta_1 = 0

local d_int_ref_d_beta_2 = `theta_3'*(`c'*exp(`beta_0' + `beta_2'*`c')/((1+exp(`beta_0' + `beta_2'*`c'))^2))

local d_int_med_d_theta_0 = 0

local d_int_med_d_theta_1 = 0

local d_int_med_d_theta_2 = 0

local d_int_med_d_theta_3 = (exp(`beta_0' + `beta_1' + `beta_2'*`c')/(1 + exp(`beta_0' + `beta_1' + `beta_2'*`c'))) - (exp(`beta_0' + `beta_2'*`c')/(1 + exp(`beta_0' + `beta_2'*`c')))

local d_int_med_d_theta_4 = 0

local d_int_med_d_beta_0 = `theta_3'*((exp(`beta_0' + `beta_1' + `beta_2'*`c')/((1+exp(`beta_0' + `beta_1' + `beta_2'*`c'))^2)) - (exp(`beta_0' + `beta_2'*`c')/((1+exp(`beta_0' + `beta_2'*`c'))^2)))

local d_int_med_d_beta_1 = `theta_3'*(exp(`beta_0' + `beta_1' + `beta_2'*`c')/((1+exp(`beta_0' + `beta_1' + `beta_2'*`c'))^2))

local d_int_med_d_beta_2 = `theta_3'*((`c'*exp(`beta_0' + `beta_1' + `beta_2'*`c')/((1+exp(`beta_0' + `beta_1' + `beta_2'*`c'))^2)) - (`c'*exp(`beta_0' + `beta_2'*`c')/((1+exp(`beta_0' + `beta_2'*`c'))^2)))

local d_pie_d_theta_0 = 0

local d_pie_d_theta_1 = 0

local d_pie_d_theta_2 = ((exp(`beta_0' + `beta_1' + `beta_2'*`c')/(1 + exp(`beta_0' + `beta_1' + `beta_2'*`c'))) - (exp(`beta_0' + `beta_2'*`c')/(1 + exp(`beta_0' + `beta_2'*`c'))))

local d_pie_d_theta_3 = 0

local d_pie_d_theta_4 = 0

local d_pie_d_beta_0 = `theta_2'*((exp(`beta_0' + `beta_1' + `beta_2'*`c')/((1+exp(`beta_0' + `beta_1' + `beta_2'*`c'))^2)) - (exp(`beta_0' + `beta_2'*`c')/((1+exp(`beta_0' + `beta_2'*`c'))^2)))

local d_pie_d_beta_1 = `theta_2'*(exp(`beta_0' + `beta_1' + `beta_2'*`c')/((1+exp(`beta_0' + `beta_1' + `beta_2'*`c'))^2))

local d_pie_d_beta_2 = `theta_2'*((`c'*exp(`beta_0' + `beta_1' + `beta_2'*`c')/((1+exp(`beta_0' + `beta_1' + `beta_2'*`c'))^2)) - (`c'*exp(`beta_0' + `beta_2'*`c')/((1+exp(`beta_0' + `beta_2'*`c'))^2)))

local d_te_d_theta_0 = `d_cde_d_theta_0' + `d_int_ref_d_theta_0' + `d_int_med_d_theta_0' + `d_pie_d_theta_0'

local d_te_d_theta_1 = `d_cde_d_theta_1' + `d_int_ref_d_theta_1' + `d_int_med_d_theta_1' + `d_pie_d_theta_1'

local d_te_d_theta_2 = `d_cde_d_theta_2' + `d_int_ref_d_theta_2' + `d_int_med_d_theta_2' + `d_pie_d_theta_2'

local d_te_d_theta_3 = `d_cde_d_theta_3' + `d_int_ref_d_theta_3' + `d_int_med_d_theta_3' + `d_pie_d_theta_3'

local d_te_d_theta_4 = `d_cde_d_theta_4' + `d_int_ref_d_theta_4' + `d_int_med_d_theta_4' + `d_pie_d_theta_4'

local d_te_d_beta_0 = `d_cde_d_beta_0' + `d_int_ref_d_beta_0' + `d_int_med_d_beta_0' + `d_pie_d_beta_0'

local d_te_d_beta_1 = `d_cde_d_beta_1' + `d_int_ref_d_beta_1' + `d_int_med_d_beta_1' + `d_pie_d_beta_1'

local d_te_d_beta_2 = `d_cde_d_beta_2' + `d_int_ref_d_beta_2' + `d_int_med_d_beta_2' + `d_pie_d_beta_2'

foreach x in theta_0 theta_1 theta_2 theta_3 theta_4 beta_0 beta_1 beta_2 {

local d_prop_cde_d_`x' = (`d_cde_d_`x''*`te' - `cde'*`d_te_d_`x'')/(`te'^2)

local d_prop_int_ref_d_`x' = (`d_int_ref_d_`x''*`te' - `int_ref'*`d_te_d_`x'')/(`te'^2)

local d_prop_int_med_d_`x' = (`d_int_med_d_`x''*`te' - `int_med'*`d_te_d_`x'')/(`te'^2)

local d_prop_pie_d_`x' = (`d_pie_d_`x''*`te' - `pie'*`d_te_d_`x'')/(`te'^2)

local d_overall_prop_med_d_`x' = ((`d_pie_d_`x'' + `d_int_med_d_`x'')*`te' - (`pie' + `int_med')*`d_te_d_`x'')/(`te'^2)

local d_overall_p_attr_int_d_`x' = ((`d_int_ref_d_`x'' + `d_int_med_d_`x'')*`te' - (`int_ref' + `int_med')*`d_te_d_`x'')/(`te'^2)

local d_overall_prop_elim_d_`x' = ((`d_int_ref_d_`x'' + `d_int_med_d_`x'' + `d_pie_d_`x'')*`te' - (`int_ref' + `int_med' + `pie')*`d_te_d_`x'')/(`te'^2)

}

matrix df_d_theta_beta = (`d_cde_d_theta_0', `d_cde_d_theta_1', `d_cde_d_theta_2', `d_cde_d_theta_3', `d_cde_d_theta_4', `d_cde_d_beta_0', `d_cde_d_beta_1', `d_cde_d_beta_2' \ ///

`d_int_ref_d_theta_0', `d_int_ref_d_theta_1', `d_int_ref_d_theta_2', `d_int_ref_d_theta_3', `d_int_ref_d_theta_4', `d_int_ref_d_beta_0', `d_int_ref_d_beta_1', `d_int_ref_d_beta_2' \ ///

`d_int_med_d_theta_0', `d_int_med_d_theta_1', `d_int_med_d_theta_2', `d_int_med_d_theta_3', `d_int_med_d_theta_4', `d_int_med_d_beta_0', `d_int_med_d_beta_1', `d_int_med_d_beta_2' \ ///

`d_pie_d_theta_0', `d_pie_d_theta_1', `d_pie_d_theta_2', `d_pie_d_theta_3', `d_pie_d_theta_4', `d_pie_d_beta_0', `d_pie_d_beta_1', `d_pie_d_beta_2' \ ///

`d_te_d_theta_0', `d_te_d_theta_1', `d_te_d_theta_2', `d_te_d_theta_3', `d_te_d_theta_4', `d_te_d_beta_0', `d_te_d_beta_1', `d_te_d_beta_2' \ ///

`d_prop_cde_d_theta_0', `d_prop_cde_d_theta_1', `d_prop_cde_d_theta_2', `d_prop_cde_d_theta_3', `d_prop_cde_d_theta_4', `d_prop_cde_d_beta_0', `d_prop_cde_d_beta_1', `d_prop_cde_d_beta_2' \ ///

`d_prop_int_ref_d_theta_0', `d_prop_int_ref_d_theta_1', `d_prop_int_ref_d_theta_2', `d_prop_int_ref_d_theta_3', `d_prop_int_ref_d_theta_4', `d_prop_int_ref_d_beta_0', `d_prop_int_ref_d_beta_1', `d_prop_int_ref_d_beta_2' \ ///

`d_prop_int_med_d_theta_0', `d_prop_int_med_d_theta_1', `d_prop_int_med_d_theta_2', `d_prop_int_med_d_theta_3', `d_prop_int_med_d_theta_4', `d_prop_int_med_d_beta_0', `d_prop_int_med_d_beta_1', `d_prop_int_med_d_beta_2' \ ///

`d_prop_pie_d_theta_0', `d_prop_pie_d_theta_1', `d_prop_pie_d_theta_2', `d_prop_pie_d_theta_3', `d_prop_pie_d_theta_4', `d_prop_pie_d_beta_0', `d_prop_pie_d_beta_1', `d_prop_pie_d_beta_2' \ ///

`d_overall_prop_med_d_theta_0', `d_overall_prop_med_d_theta_1', `d_overall_prop_med_d_theta_2', `d_overall_prop_med_d_theta_3', `d_overall_prop_med_d_theta_4', `d_overall_prop_med_d_beta_0', `d_overall_prop_med_d_beta_1', `d_overall_prop_med_d_beta_2' \ ///

`d_overall_p_attr_int_d_theta_0', `d_overall_p_attr_int_d_theta_1', `d_overall_p_attr_int_d_theta_2', `d_overall_p_attr_int_d_theta_3', `d_overall_p_attr_int_d_theta_4', `d_overall_p_attr_int_d_beta_0', `d_overall_p_attr_int_d_beta_1', `d_overall_p_attr_int_d_beta_2' \ ///

`d_overall_prop_elim_d_theta_0', `d_overall_prop_elim_d_theta_1', `d_overall_prop_elim_d_theta_2', `d_overall_prop_elim_d_theta_3', `d_overall_prop_elim_d_theta_4', `d_overall_prop_elim_d_beta_0', `d_overall_prop_elim_d_beta_1', `d_overall_prop_elim_d_beta_2' )

matrix cov_f = (df_d_theta_beta*var_theta_beta*df_d_theta_beta')

*** Results

di "Controlled direct effect = " `cde' ", with 95% CI: [" `cde' - invnorm(0.975)*sqrt(cov_f[1,1]) ", " `cde' + invnorm(0.975)*sqrt(cov_f[1,1]) "]"

di "Reference interaction = " `int_ref' ", with 95% CI: [" `int_ref' - invnorm(0.975)*sqrt(cov_f[2,2]) ", " `int_ref' + invnorm(0.975)*sqrt(cov_f[2,2]) "]"

di "Mediated interaction = " `int_med' ", with 95% CI: [" `int_med' - invnorm(0.975)*sqrt(cov_f[3,3]) ", " `int_med' + invnorm(0.975)*sqrt(cov_f[3,3]) "]"

di "Pure indirect effect = " `pie' ", with 95% CI: [" `pie' - invnorm(0.975)*sqrt(cov_f[4,4]) ", " `pie' + invnorm(0.975)*sqrt(cov_f[4,4]) "]"

di "Total effect = " `te' ", with 95% CI: [" `te' - invnorm(0.975)*sqrt(cov_f[5,5]) ", " `te' + invnorm(0.975)*sqrt(cov_f[5,5]) "]"

di "Proportion attributable to the controlled direct effect = " `prop_cde' ", with 95% CI: [" `prop_cde' - invnorm(0.975)*sqrt(cov_f[6,6]) ", " `prop_cde' + invnorm(0.975)*sqrt(cov_f[6,6]) "]"

di "Proportion attributable to the reference interaction = " `prop_int_ref' ", with 95% CI: [" `prop_int_ref' - invnorm(0.975)*sqrt(cov_f[7,7]) ", " `prop_int_ref' + invnorm(0.975)*sqrt(cov_f[7,7]) "]"

di "Proportion attributable to the mediated interaction = " `prop_int_med' ", with 95% CI: [" `prop_int_med' - invnorm(0.975)*sqrt(cov_f[8,8]) ", " `prop_int_med' + invnorm(0.975)*sqrt(cov_f[8,8]) "]"

di "Proportion attributable to the pure indirect effect = " `prop_pie' ", with 95% CI: [" `prop_pie' - invnorm(0.975)*sqrt(cov_f[9,9]) ", " `prop_pie' + invnorm(0.975)*sqrt(cov_f[9,9]) "]"

di "Overall proportion mediated = " `overall_prop_med' ", with 95% CI: [" `overall_prop_med' - invnorm(0.975)*sqrt(cov_f[10,10]) ", " `overall_prop_med' + invnorm(0.975)*sqrt(cov_f[10,10]) "]"

di "Overall proportion attributable to interaction = " `overall_prop_attr_int' ", with 95% CI: [" `overall_prop_attr_int' - invnorm(0.975)*sqrt(cov_f[11,11]) ", " `overall_prop_attr_int' + invnorm(0.975)*sqrt(cov_f[11,11]) "]"

di "Overall proportion eliminated = " `overall_prop_elim' ", with 95% CI: [" `overall_prop_elim' - invnorm(0.975)*sqrt(cov_f[12,12]) ", " `overall_prop_elim' + invnorm(0.975)*sqrt(cov_f[12,12]) "]"

**Supplementary Table 1. Characteristics of participants included in analysis of ALSPAC data, N=2,122**

|  | Median (IQR) or N (%) |
| --- | --- |
| ***Social class*** |  |
| Low social class in childhood* | 1,185 (55.8%) |
| Low social class at mean age 30y** (entry into cohort) | 675 (31.8%) |
|  |  |
| ***Life course patterns of social class (childhood* - adulthood**)*** |  |
| Low – low | 486 (22.9%) |
| Low – high | 699 (32.9%) |
| High – low | 189 (8.9%) |
| High – high | 748 (35.3%) |
|  |  |
| ***Assessment of physical capability in mid-life*** |  |
| Age (years) | 50.7 (48.1 to 53.6) |
| Not able to perform handgrip test | 11 (0.5%) |
| Not able to perform chair rise test | 42 (2.0%) |
| Not able to perform one-legged test | 11 (0.5%) |
| Not able to perform 3 metre timed walk unaided | 11 (0.5%) |
| Maximum handgrip (kg)*** | 26 (22 to 30) |
| Chair rise time (seconds) | 22.5 (19.7 to 26) |
| One legged stand (seconds)**** | 4.8 (3.0 to 9.8) |
| Timed 3 metre walk (seconds) | 2.4 (2.2 to 2.7) |

* Defined by paternal occupation, using the Registrar General’s Social Classes; high social class is defined as categories I and II, low social class is defined as categories III non-manual and below

** Defined by highest of own or partner’s occupation, using the Registrar General’s Social Classes; high social class is defined as categories I and II, low social class is defined as categories III non-manual and below

*** Maximum value of 4 measures (2 in each hand)

**** With eyes closed, up to a maximum of 30 seconds
