# Supplementary material for: Relationship between mediation analysis and the structured life course approach
Source: Int J Epidemiol. 2016 Oct 6;45(4):1280–94. doi: 10.1093/ije/dyw254 (PMC5841634; doi:10.1093/ije/dyw254)
Supplement: Supplementary Data [file dyw254_supplementary_data.zip › Appendix.pdf]

Appendix: Derivation of relationship between decomposition analyses and life course models

VanderWeele(12) showed that, under certain conditions, when the exposure and mediator are both binary variables, the 4-way decomposition in **Box 1** estimates the following components, which are reproduced here using counterfactual notation:

CDE(0)	$Y_{10} - Y_{00,}$
INT_{ref}	$(Y_{11} - Y_{10} - Y_{01} + Y_{00})\, M_{0,}$
INT_{med}	$(Y_{11} - Y_{10} - Y_{01} + Y_{00})\, (M_1 - M_0)$,
PIE	$(Y_{01} - Y_{00})\, (M_1 - M_0)$,

Y_{xm} is the value of the outcome (Y) if the exposure (X) were set to *x* and mediator (M) were set to *m*, and M_x is the value of the mediator if the exposure were set to *x*.

The seven life course models in **Box 2** can be summarized by how the potential outcome differs between levels of the exposure and mediator:

Early life critical period	$Y_{00} = Y_{01} \neq Y_{10} = Y_{11}$
Adult critical period	$Y_{00} = Y_{10} \neq Y_{01} = Y_{11}$
Accumulation	$Y_{01} = Y_{10},\ Y_{11} - Y_{10} = Y_{01} - Y_{00} \neq 0$
Increasing social class	$Y_{10} \neq Y_{00} = Y_{01} = Y_{11}$
Decreasing social class	$Y_{01} \neq Y_{00} = Y_{10} = Y_{11}$
Always exposed	$Y_{00} = Y_{01} = Y_{10} \neq Y_{11}$
Ever exposed	$Y_{00} \neq Y_{01} = Y_{10} = Y_{11}$

Using these equalities and inequalities, we can quickly see which of the components of the 4-way decomposition are non-zero.

The **controlled direct effect** will be non-zero when $Y_{10} - Y_{00} \neq 0$. Therefore the *early life critical period*, *accumulation*, *increasing social class* and *ever exposed* models will show a controlled direct effect.

The **pure indirect effect** can only be non-zero if $Y_{01} – Y_{00} \neq 0$, which happens under the *adult critical adulthood*, *accumulation*, *decreasing social class* and *ever exposed* models. Therefore a pure indirect effect will show in these models provided that $M_1 – M_0 \neq 0$, i.e. there is an association between exposure and mediator.

The **reference interaction** will be non-zero if $Y_{11} – Y_{10} – Y_{01} + Y_{00} \neq 0$, under the reasonable assumption that $M_0 \neq 0$. Therefore the *increasing social class*, *decreasing social class*, *always exposed* and *ever exposed* models will show a reference interaction.

The **mediated interaction** can only be non-zero if $Y_{11} – Y_{10} – Y_{01} + Y_{00} \neq 0$, which occurs under the *increasing social class*, *decreasing social class*, *always exposed* and *ever exposed* models. Therefore a mediated interaction will show in these models provided that $M_1 – M_0 \neq 0$.

The **total indirect effect** is TIE = PIE + INT_{med}. In general, this will be non-zero if PIE and/or INT_{med} are non-zero. Therefore a total indirect effect will show in the *critical adulthood*, *accumulation*, *increasing social class* and *always exposed* models, provided that $M_1 – M_0 \neq 0$. However, in the decreasing social class and ever exposed models, we have $Y_{11} – Y_{10} = 0$ and hence PIE = $–\text{INT}_{\text{med}}$, so a total indirect effect will not show in these models in spite of having non-zero PIE and non-zero INT_{med}.

To consider the conventional direct and indirect effects in mediation analysis, we switch from counterfactuals to regression models. Suppose then that the outcome can be modelled by the linear model with interaction:

$$Y = \alpha + \beta X + \gamma M + \delta XM + \varepsilon,$$

where $E(\varepsilon) = 0$ and ε is independent of the binary X and M. The seven life course models in **Box 2** can be summarized by the relationships of the regression parameters β, γ and δ:

Early life critical period	$\beta \neq 0, \gamma = 0, \delta = 0$
Adult critical period	$\beta = 0, \gamma \neq 0, \delta = 0$
Accumulation	$\beta = \gamma \neq 0, \delta = 0$
Increasing social class	$\beta = -\delta \neq 0, \gamma = 0$
Decreasing social class	$\beta = 0, \gamma = -\delta \neq 0$
Always exposed	$\beta = 0, \gamma = 0, \delta \neq 0$
Ever exposed	$\beta = \gamma = -\delta \neq 0$

The conventional **Direct Effect** is the regression coefficient of Y on X, adjusted for M. This can be calculated using a standard formula:

$$(\text{var}(M)\ \text{cov}(X,Y) - \text{cov}(X,M)\ \text{cov}(M,Y)) / (\text{var}(M)\ \text{var}(X) - \text{cov}(X,M)^2).$$

The covariances with the outcome are

$$\begin{aligned}\text{cov}(X,Y) &= \text{cov}(X, \alpha + \beta X + \gamma M + \delta XM + \varepsilon)\\ &= \text{cov}(X,\alpha) + \text{cov}(X,\beta X) + \text{cov}(\underline{X},\gamma M) + \text{cov}(X,\delta XM) + \text{cov}(X,\varepsilon)\\ &= \beta\ \text{var}(X) + \gamma\ \text{cov}(X,M) + \delta\ \text{cov}(X,XM)\end{aligned}$$

and

$$\begin{aligned}\text{cov}(M,Y) &= \text{cov}(M, \alpha + \beta X + \gamma M + \delta XM + \varepsilon)\\ &= \text{cov}(M,\alpha) + \text{cov}(M,\beta X) + \text{cov}(M,\gamma M) + \text{cov}(M,\delta XM) + \text{cov}(M,\varepsilon)\\ &= \beta\ \text{cov}(X,M) + \gamma\ \mathit{var}(M) + \delta\ \text{cov}(M,XM).\end{aligned}$$

Therefore the numerator in the direct effect formula is $\beta\ \text{var}(M)\ \text{var}(X) + \gamma\ \text{var}(M)\ \text{cov}(X,M) + \delta\ \text{var}(M)\ \text{cov}(X,XM) - \beta\ \text{cov}(X,M)^2 - \gamma\ \text{var}(M)\ \text{cov}(X,M) - \delta\ \text{cov}(X,M)\ \text{cov}(M,XM)$. The direct effect is consequently

$$\beta + \delta\ (\text{var}(M)\ \text{cov}(X,XM) - \text{cov}(X,M)\ \text{cov}(M,XM)) / (\text{var}(M)\ \text{var}(X) - \text{cov}(X,M)^2).$$

The fact that X and M are binary means that $\text{var}(X) = E(X)(1-E(X))$ and $\text{var}(M) = E(M)(1-E(M))$. Furthermore $E(XM) = E(X^2M) = E(XM^2)$, so that $\text{cov}(X,XM) = E(X^2M)-E(X)E(XM) = (1-E(X))E(XM)$ and $\text{cov}(M,XM) = E(XM^2)-E(M)E(XM) = (1-E(M))E(XM)$. To see that the

coefficient for δ in the direct effect is non-zero, note that substituting the above expressions into var(M) cov(X,XM) – cov(X,M) cov(M,XM) gives (1–E(M))E(XM)(E(M)–E(XM)), which can only be zero if E(M) = 1 or E(XM) = 0, or if E(XM) = E(M). These conditions cannot happen if all combinations of X and M occur with non-zero probability. It is possible that the two terms in the direct effect will cancel each other by chance. This is a disadvantage that is not present in the 4 way decomposition. Ignoring this possibility, it is easier to identify the models in which the direct effect is zero. This will occur if both $\beta = 0$ and $\delta = 0$. The only model for which this is the case, and there is no direct effect, is the *adult critical period* model.

The conventional **Indirect Effect** is the product of the regression coefficient of Y on M, adjusted for X, and the regression coefficient of M on X. The adjusted regression coefficient is

$$(\mathrm{var}(X)\ \mathrm{cov}(M,Y) - \mathrm{cov}(X,M)\ \mathrm{cov}(X,Y)) / (\mathrm{var}(M)\ \mathrm{var}(X) - \mathrm{cov}(X,M)^2),$$

with numerator β var(X) cov(X,M) + γ var(X) var(M) + δ var(X) cov(M,XM) – β var(X) cov(X,M) – γ cov(X,M)^2 – δ cov(X,M) cov(X,XM). The regression coefficient of M on X is cov(X,M) / var(X), and the indirect effect is therefore

$$(\mathrm{cov}(X,M) / \mathrm{var}(X))\ (\gamma + \delta\ (\mathrm{var}(X)\ \mathrm{cov}(M,XM) - \mathrm{cov}(X,M)\ \mathrm{cov}(X,XM)) / (\mathrm{var}(M)\ \mathrm{var}(X) - \mathrm{cov}(X,M)^2)).$$

The coefficient for δ is (1–E(X))E(XM)(E(X)–E(XM)), which once again cannot be zero if all combinations of X and M occur with non-zero probability. The indirect effect will always be zero unless cov(X,M) $\neq 0$, i.e. there is an association between exposure and mediator. Again ignoring chance cancellation, it is simpler to show the models in which the indirect effect is zero. This is only certain to occur when $\gamma = 0$ and $\delta = 0$. Only in the *early critical period* model will this occur and there be no Indirect Effect.

Some life course models can be thought of as a combination of simpler models. For instance a ‘sensitive period’ hypothesis can be thought of as a combination of a critical period and accumulation models. Should such a model be identified, all effects that are non-zero in the component models could be considered to be non-zero in the identified compound model. There is the possibility that the effects of the separate component models may cancel each other out by chance.
