# Supplementary material for: Relationship between mediation analysis and the structured life course approach
Source: Int J Epidemiol. 2016 Oct 6;45(4):1280–94. doi: 10.1093/ije/dyw254 (PMC5841634; doi:10.1093/ije/dyw254)
Supplement: Supplementary Data [file supp_45_4_1280__index.html]

Relationship between mediation analysis and the structured life course approach — Supplementary Data 

# Relationship between mediation analysis and the structured life course approach

## Supplementary Data

files

- Supplementary Data - docx file
- Supplementary Data - pdf file
